# PSDSD-A Superpixel Generating Method Based on Pixel Saliency Difference and Spatial Distance for SAR Images

**DOI:** 10.3390/s19020304

**Published:** 2019-01-14

**Authors:** Tao Xie, Jingjian Huang, Qingzhan Shi, Qingping Wang, Naichang Yuan

**Affiliations:** State Key Laboratory of Complex Electromagnetic Environment Effects on Electronics and Information System, National University of Defense Technology, Sanyi Avenue, Kaifu District, Changsha 410073, Hunan, China; xietao09@nudt.edu.cn (T.X.); qingzhanshi@foxmail.com (Q.S.); andywpq007@163.com (Q.W.); yuannaichang@hotmail.com (N.Y.)

**Keywords:** superpixel, synthetic aperture radar (SAR) image, visual saliency, local contrast measure, Gaussian kernel function

## Abstract

Superpixel methods are widely used in the processing of synthetic aperture radar (SAR) images. In recent years, a number of superpixel algorithms for SAR images have been proposed, and have achieved acceptable results despite the inherent speckle noise of SAR images. However, it is still difficult for existing algorithms to obtain satisfactory results in the inhomogeneous edge and texture areas. To overcome those problems, we propose a superpixel generating method based on pixel saliency difference and spatial distance for SAR images in this article. Firstly, a saliency map is calculated based on the Gaussian kernel function weighted local contrast measure, which can not only effectively suppress the speckle noise, but also enhance the fuzzy edges and regions with intensity inhomogeneity. Secondly, superpixels are generated by the local *k*-means clustering method based on the proposed distance measure, which can efficiently sort pixels to different clusters. In this step, the distance measure is calculated by combining the saliency difference and spatial distance with a proposed adaptive local compactness parameter. Thirdly, post-processing is utilized to clean up small segments. The evaluation experiments on the simulated SAR image demonstrate that our proposed method dramatically outperforms four state-of-the-art methods in terms of boundary recall, under-segmentation error, and achievable segmentation accuracy under almost all of the experimental parameters at a moderate segment speed. The experiments on real-world SAR images of different sceneries validate the superiority of our method. The superpixel results of the proposed method adhere well to the contour of targets, and correctly reflect the boundaries of texture details for the inhomogeneous regions.

## 1. Introduction

Synthetic aperture radars (SAR) have the key characteristics of all-day all-weather observation and strong surface penetration capabilities, which play an important role in the field of remote sensing [1]. SAR images are widely used across numerous fields. For example, military applications include target detection, precision guidance, and impact assessment, and the civil areas include terrain mapping, crop growth assessment, and environmental and disaster monitoring [2,3,4,5,6,7,8,9,10]. With the development of radar satellite technology, the collected radar image data shows blowout growth. Traditional pixel-based image analysis (PBIA) methods are increasingly difficult to meet people’s needs for data processing. In order to adapt to the new situation, object-based image analysis (OBIA) methods have been proposed and become a new paradigm [11,12,13]. The OBIA methods can not only meet people’s real-time demand for big data processing, but also pave the way for the application of artificial intelligence, machine learning, and other new technologies [14,15]. Superpixels refer to the irregular block of pixels with certain visual significance composed of adjacent pixels with similar texture, color, brightness, and other characteristics [16]. In recent years, a large number of superpixel algorithms have been proposed. For example, Mean Shift [17], Quick Shift [18], the Watershed approach [19], the Turbopixel method [20], the Normalized-Cuts [21] and the SEEDS (Superpixels Extracted via Energy-Driven Sampling) [22] et al. However, these methods are not designed for SAR images, and they may not perform well in SAR images due to the inherent speckles [23,24].

In 2012, Achanta et al. [25] proposed a simple linear iterative clustering (SLIC) method to generate superpixels for optical images. The SLIC calculates the spatial distance and the color distance between pixels and cluster centers, then the local *k*-means clustering (LKMC) method is implemented to generate superpixels. The SLIC method performs well in optical images, but the resulting superpixels are unsatisfying when applied to SAR images. Then, several superpixel methods based on SLIC were introduced to perform superpixel segmentation in SAR images. In 2013, Xiang et al. [26] devised a superpixel generating algorithm based on pixel intensity and location similarity (PILS) for SAR images. They defined the pixel intensity similarity, which is robust for speckle noise; then, the pixel intensity and location similarities were used to generate superpixels. In 2016, Zou et al. [27] proposed a likelihood-based SLIC (LBSLIC) superpixel algorithm for SAR images. They believed that the likelihood information instead of the mean intensity of SAR image clusters is more useful due to the inherent speckle noise; thus, the likelihood value rather than the Euclidean distance was adopted to represent the intensity similarity between a pixel and a cluster. In the same year, Yu et al. [28] introduced a new method to evaluate the dissimilarity of two pixels by measuring the dissimilarity of the two local patches centering these two pixels, which called the patch-based SLIC (PBSLIC). They thought that two local patches instead of two pixels are more effective to suppress the speckles in SAR images. These methods above are all based on the SLIC algorithm, and improved the distance measurement to adapt to the speckle noise in SAR images. These methods have achieved munch better segmentation results than SLIC; however, the results are still unsatisfactory in edge regions and inhomogeneous areas.

We studied the reason for bad superpixels in inhomogeneous regions, and found that it is the dissimilarity instead of the measurement that restricts the accuracy of classification when generating superpixels. That is to say, pixels in inhomogeneous regions and texture areas share a narrow range of pixel intensity, which caused the failure judgement when sorting pixels to different clusters. Therefore, we think it would be useful to enhance the contrast between pixels of inhomogeneous regions, which would enlarge the dynamic range of these pixels and make it easier to sort these pixels into different superpixels.

As vision scientists concluded, contrast is the most important quantity encoded in the streams of the visual system. Chen et al. [29] introduced the basic concept of local contrast measure (LCM) inspired by the robust properties of the human vision system (HVS) and derived kernel (DK) model [30]. By the LCM method, target signal enhancement and background clutter suppression are achieved simultaneously. Based on this characteristic, we can solve the problem that bad superpixel results are generated in inhomogeneous regions and fuzzy edge areas.

In this article, we innovatively introduce the visual saliency into the distance measurement, and a superpixel-generating method is proposed based on pixel saliency difference and spatial distance (PSDSD). Firstly, the saliency map of the SAR image is calculated based on the Gaussian kernel function weighted local contrast measure (GLCM). Secondly, the local *k*-means clustering method based on our distance measure is implemented to generate superpixel results. The distance measure is produced by combining the saliency difference and spatial distance with an adaptive local compactness (ALC) parameter. Finally, a post-processing procedure is utilized to clean up the small segments.

This article is organized as follows. In Section 2, the existing superpixel methods for SAR images are presented, and the LCM is introduced and outlined. The proposed pixel saliency difference and spatial distance algorithm is described in Section 3. The experimental results and analysis are given in Section 4. The conclusion is in Section 5.

## 2. Related Work

### 2.1. Existing Superpixel Methods for SAR Images

Since the SLIC [25] method was proposed, scholars who study SAR images devised a number of superpixel generating algorithms, such as PILS [26], LBSLIC [27], and PBSLIC [28], based on the core idea of the SLIC method.

**Figure 1:**
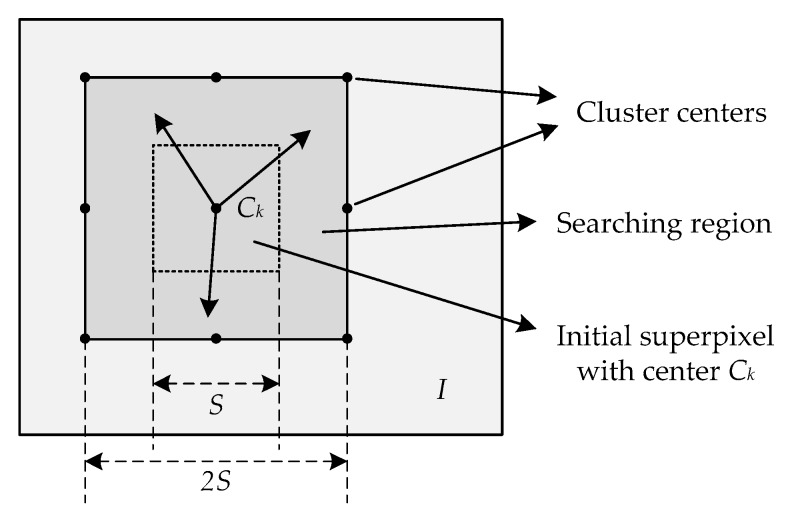
The searching region of local *k*-means clustering (LKMC).

The core idea of SLIC is local *k*-means clustering (LKMC), which has been proved to have more computational efficiency than the conventional *k*-means algorithm [25]. As shown in Figure 1, let the number of total pixels of the image *I* be *N*, and the number of superpixels to be segmented be *K*, to produce roughly equally-sized superpixels; the grid interval is $S=\sqrt{N/K}$. The detailed processing flow of the LKMC is as follows [25]:

*Step* *1:* *Initialization*
  Initialize cluster centers *C_k_* (*l_k_*, *I_k_*, *x_k_*, *y_k_*) by sampling pixels at regular grid steps *S*.
  Maximum iteration set to *M*.
  Set Iter = 0.
  Set label map *l*(*i*) = −1 for each pixel *i*.
  Set distance map *d*(*i*) = ∞ for each pixel *i*.
*Step* *2:* *Assignment*
  For each cluster center *C_k_*, search each pixel *i* in a 2*S* × 2*S* local search region and compute the distance *D* between *C_k_* and *i*.
  If *D* < *d*(*i*), then set *d*(*i*) = *D* and set *l*(*i*) = *k*.
*Step* *3:* *Update cluster centers*
  Compute new cluster center *C_k_* (*l_k_*, *I_k_*, *x_k_*, *y_k_*) for each cluster.
  Iter = Iter + 1.
*Step* *4:* *Repeat Step 2–3*
  Do *Step 2*–*3* while Iter < *M*.

In *Step 1*, *l_k_*, *I_k_*, *x_k_*, and *y_k_* are the label of *C_k_*, the intensity of cluster center *C_k_*, and the *x*-coordinate and *y*-coordinate of cluster center *C_k_*, respectively. In *Step 3*, *l_k_*, *I_k_*, *x_k_*, and *y_k_* are the label of *C_k_*, the mean intensity of pixels in cluster *C_k_*, and the mean values of the *x*-coordinate and *y*-coordinates of the pixels in cluster *C_k_*, respectively.

In *Step 2*, the distance measure *D* is utilized to sort pixels into different superpixels. The distance measure is different from one to another for SLIC, PILS, LBSLIC, and PBSLIC. Basically, these distance measures are all based on the distance or dissimilarity of two pixels or two patches [25,26,27,28]. However, pixels in inhomogeneous regions and texture areas share a narrow range of intensities, and methods based on the distance or dissimilarity mechanism would be insensitive to these slight changes of intensities, and lead to bad superpixel results under this situation.

### 2.2. The Local Contrast Measure

Chen et al. [29] proposed the local contrast measure (LCM) to obtain the local contrast intensity map of the image by calculating the difference between the pixel and the neighboring region in order to enhance weak and small targets and suppress the clutter background for the infrared image. The LCM method uses visual saliency to significantly enhance the weak and small targets in the image.

**Figure 2:**
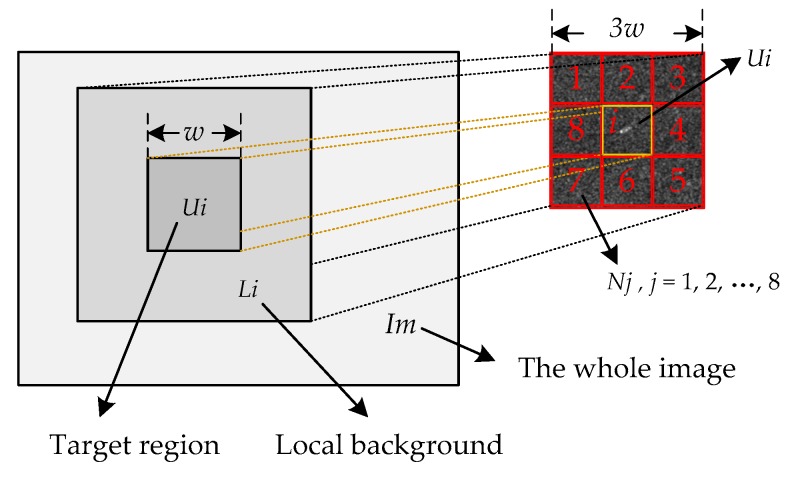
Local region and local background window.

As is shown in Figure 2, *U_i_* denotes a *w* × *w* size target region around pixel *i*. *L_i_* denotes the local background region of *U_i_*, and consists of eight smaller regions *N_j_*, *j* = 1, 2, …,8. We denote *m_j_* to be the mean gray scale of each near region *N_j_*, then:

(1) $$m_{j}=mean(I),I\in N_{j}.$$

Denote *V_i_* as the maximum gray scale of local region *U_i_*, then:

(2) $$V_{i}=\max(I),I\in U_{i}.$$

Thus, the contrast between the local region *U_i_* and the near region *N_j_* can be deserved as:

(3) $$c_{i}^{j}=\frac{V_{i}}{m_{j}},j=1,2,\ldots ,8.$$

Considering the pixel *i* belongs to the target region, then the contrast measure should be dramatic. Thus, the local contrast measure (LCM) is calculated as:

(4) $$S_{i}=\underset{j}{\min}V\times c_{i}^{j},j=1,2,\ldots ,8.$$

Denote *M_i_* as the maximum value of *m_j_*, *j* = 1, 2, 3, …, 8. Then, the LCM of pixel *i* can be described as:

(5) $$S_{i}=V_{i}\times \frac{V_{i}}{M_{i}}=\frac{V_{i}^{2}}{M_{i}}.$$

**Figure 3:**
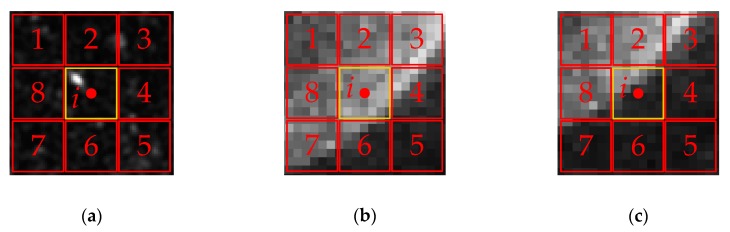
(**a**) The image patches with speckle; (**b**) The center pixel locates to the brighter side of the local region; (**c**) The center pixel locates to the darker side of the local region.

As for the small target region, the LCM method can significantly enhance the target, especially for the targets that have the same size as the local region size. While, as is shown in Figure 3a, if the speckle appears in the local region, the LCM method will also strengthen the noise, which is not conducive to SAR image processing.

In addition, the LCM method shows the inappropriate handling of marginal areas. As is shown in Figure 3b, when the pixel *i* locates to the brighter side of the edge region, the minimal value of contrast operator $c_{i}^{j}$ is about equal to one, and *V_i_* is also about equal to the value of pixel *i*; thus, the LCM operator has little effect on pixel *i* under this situation. As is shown in Figure 3c, when the pixel *i* locates to the darker side of the edge region, the operator $c_{i}^{j}$ is also about equal to one, but *V_i_* is dramatically larger than the value of pixel *i*. Then, the pixel *i* is enhanced by the LCM operator, which should have been weakened to highlight the boundary.

## 3. Proposed Method

### 3.1. Gaussian Kernel Weighted Local Contrast Measure

Taking into account the drawbacks of the LCM, we proposed a Gaussian kernel weighted LCM (GLCM) method. We introduce the standard Gaussian kernel function, which has been proven to be robust to multiplicative noise [31], to calculate the equivalent value of pixel *i*. As shown is Figure 2, *N* is the number of pixels in region *U_i_*, and *G_i_* denotes the equivalent value of pixel *i*, then:

(6) $$G_{i}=\sum_{k=1}^{N}G(k)\times I(k),I\in U_{i}.$$

Thus, the saliency intensity of pixel *i* can be derived by our GLCM method as:

(7) $$S_{i}=V_{i}\times \frac{G_{i}}{M_{i}}.$$

By our GLCM method, the calculation of the saliency intensity is more effective compared with the LCM method.

First, for the speckle noise situation, the proposed measure *G_i_* is superior to *V_i_* because:

1. The *V_i_* uses the maximum value of the local region, which would dramatically enlarge the speckle noise.
2. *G_i_* is neither the value of pixel *i*, nor the value of the speckle noise; it is an equivalent value of the local region calculated by the standard Gaussian kernel, which would efficiently suppress the speckle.

Second, for the marginal situation, as is shown in Figure 3b, when the pixel *i* locates to the brighter side of the edge, *V_i_* is about equal to *M_i_*, but most of the pixels of the local region *U_i_* are brighter than the pixel *i*, so, the *G_i_* is larger than the value of pixel *i*. Then, the GLCM operator enhances the pixels, which are located on the brighter side of the edge. As for Figure 3c, the pixel *i* is located on the darker side of the edge; then, *V_i_* is also about equal to *M_i_*, but most of the pixels of the local region *U_i_* are darker than pixel *i*; thus, the GLCM operator can weaken the pixels, which are located on the darker side of the edge.

**Figure 4:**
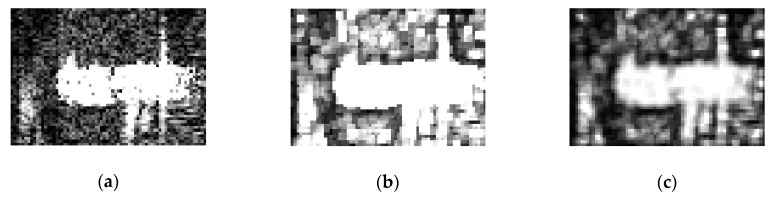
(**a**) Original synthetic aperture radar (SAR) image; (**b**) Saliency map by the local contrast measure (LCM); (**c**) Saliency map by Gaussian kernel function weighted local contrast measure (GLCM).

Figure 4a is a SAR image corrupted by speckle noise, which displays a ship target in the sea. Figure 4b,c show the saliency map of the original image by the LCM and GLCM methods (transformed to the eight-bit single channel image), respectively. Obviously, the ship is drowning in speckles, and is hard to recognize, as is shown in Figure 4b, because the LCM method dramatically amplifies noises when trying to make the target more significant. However, one benefit from the Gaussian kernel function, which is robust to speckles, is that the body outlines in Figure 4c are clearly recognizable, and the speckles are suppressed effectively by our GLCM method.

**Figure 5:**
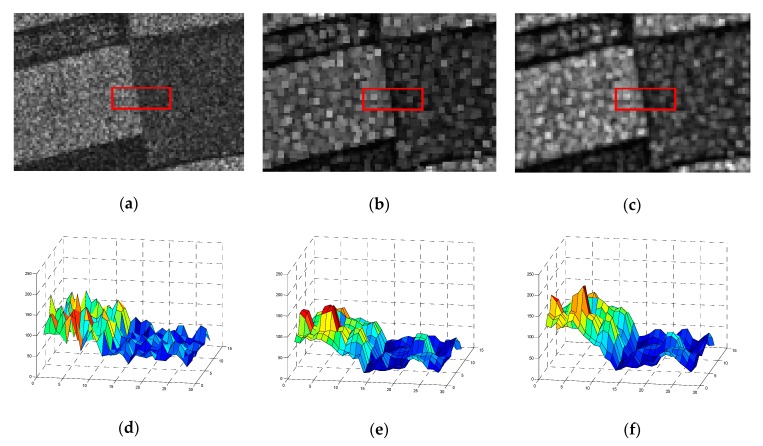
(**a**) Original SAR image; (**b**) Saliency map by LCM; (**c**) Saliency map by GLCM; (**d**–**f**) 3-D surface intensity map of regions in red rectangle: (**d**) The original map; (**e**) Saliency map by LCM; (**f**) Saliency map by GLCM.

As we can see in Figure 5, the original SAR image is a part of the farmland with several types of crops. The rectangle in red circles some cross-region of two crop fields. We can visually recognize the fuzzy edge of these two fields; however, it is difficult for traditional distance measures to distinguish them. Figure 5d shows the 3-D surface view of the intensities of pixels in the original image, and it can be seen that the intensities of these two fields are so similar that it’s challenging to tell them apart. Figure 5b,c are the saliency map by LCM and GLCM, and Figure 5e,f are the 3-D surface view of these two images, respectively. We can see that dynamic range of the intensities of the pixels is enlarged by both the LCM and GLCM methods. However, as we can see, the results by our GLCM method are more significant than those of the LCM method. Especially in the edge areas, the gap of pixel intensity between the two fields are dramatically enlarged, which makes it easier to distinguish them from each other.

In summary, our GLCM method can not only suppress the impact of speckle noise, but also effectively enhance the contrast of the marginal areas, which provides a favorable condition for the superpixel generation of inhomogeneous SAR images.

### 3.2. Adaptive Local Compactness Parameter

In traditional methods, a constant compactness parameter is adopted to weigh the relative importance between pixel intensities and spatial proximity [25,26,27,28]. However, in images with hybrid scenes, a constant regularization parameter would not be suitable, and it is more reasonable to use different compactness parameters according to the contents of the image areas.

For the areas with uniform pixel intensities, the spatial distance outweighs the intensity difference; then, more relative importance should be given to the spatial distance than the intensity difference. While, for the regions rich in feature information, the spatial distance should be less important than the intensity distance to highlight the textures and boundaries of the image. Thus, we proposed an adaptive local compactness (ALC) parameter based on a global regularization parameter. The ALC is defined as follows:

(8) $$m_{c}=\exp(-\left|S_{i}-S_{j}\right|),m_{c}\in (0,1],$$

*S_i_* and *S_j_* are the saliency intensities of pixel *i* and *j*, respectively. We can derive the regularization parameter as:

(9) $$m=m_{g}\cdot m_{c}=m_{g}\exp(-\left|S_{i}-S_{j}\right|),$$

where *m_g_* is the global compactness parameter, which is used to control the overall regularization of the resulting superpixels.

In homogeneous areas, the saliency intensities of pixel *i* and *j* tend to be the same; thus, *m_c_* tends to be one, which means that the regularity of resulting superpixels are mainly decided by the global compactness parameter. While for regions rich in feature information such as buildings and roads, the saliency intensities cover a large dynamic range, and the larger the saliency difference between pixel *i* and *j*, the smaller the *m_c_*; thus the global regularization parameter *m_g_* is further weakened.

**Figure 6:**
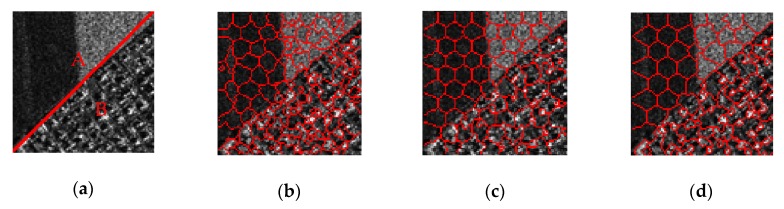
Superpixel results with different compactness parameters in hybrid regions: (**a**) Original SAR image of hybrid regions A and B; (**b**) Results with constant compactness parameter *m* set to five; (**c**) Results with constant compactness parameter *m* set to 10; (**d**) Results by adaptive local compactness (ALC) with the global compactness parameter *m_g_* set to 10.

As we can see in Figure 6a, this SAR image shows the border area between residential areas and the farmland, and we divide the image into two parts, A and B, along the diagonal line. Figure 6b,c show the superpixel results with constant compactness parameters *m* set to five and 10, respectively. We can see that over-segmentations occur when a relatively smaller *m* is adopted for region A. Meanwhile, the resulting superpixels failed to adhere to the boundaries of the buildings when a relatively larger *m* is adopted for region B.

The result of our proposed ALC method with *m_g_* set to 10 is shown in Figure 6d, and we can see that the regular results are generated in region A, and at the same time, the superpixels that adhere well to the contours of our targets such as buildings and roads are provided in region B.

In hybrid scenes, we hope that the resulting superpixels in uniform regions be more regular, while they also adhere more to contours of targets in regions containing buildings, roads, and other architectures. In this way, traditionally methods using a constant compactness parameter would not produce approving results, and it is also inappropriate to tune it manually according to the image. Thus, we adopt an adaptive local compactness parameter to produce better superpixels for SAR images.

### 3.3. The Proposed Distance Measure and Processing Flow

In this article, we utilize the LKMC to generate superpixels, due to the the high computationally efficiency of the LKMC method [25]. Based on the GLCM and the ALC, the distance measure of our proposed PSDSD is devised as follows:

(10) $$D(i,j)=D_{s}(i,j)+m\cdot D_{xy}(i,j),$$

where *D_s_* is the saliency difference between pixel *i* and *j*, which is defined as:

(11) $$D_{s}(i,j)=\left|S_{i}-S_{j}\right|,$$

and the spatial distance *D_xy_* is defined as:

(12) $$D_{xy}(i,j)=\sqrt{{\left(x_{i}-x_{j}\right)}^{2}+{\left(y_{i}-y_{j}\right)}^{2}}.$$

and the regularization parameter *m* was defined in Equation (9).

**Figure 7:**
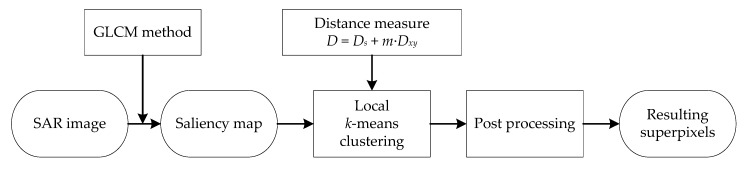
The flow chart of the proposed pixel saliency difference and spatial distance (PSDSD) method.

Then, the processing flow of our proposed PSDSD algorithm is shown in Figure 7.

Firstly, the saliency map of the input SAR image is calculated by our proposed GLCM method. Actually, the saliency intensity of each pixel is derived from the GLCM operator; then, we normalize the intensity map, and after that, we transform the intensity map into the saliency map, which is an eight-bit single channel image.

Secondly, LKMC is utilized to generate superpixel segmentations based on the proposed distance measure. The processing flow of LKMC has been described in Section 2.1. Unlike traditional algorithms, we adopt the proposed saliency difference and spatial distance measure in the assignment step to sort pixels into different clusters.

Finally, a post-processing procedure is implemented to clean up small segments. In this article, the orphaned segments, which contain less than three pixels, are assigned to the nearest cluster center.

## 4. Experiments and Analysis

In this section, experiments are implemented to our proposed method, and four state-of-the-art superpixel methods, i.e., SLIC, PILS, LBSLIC, and PBSLIC on both simulated SAR images and real-world SAR images. The performances of these algorithms were tested using MATLAB software on a computer equipped with a 2.6 GHz Intel i7 processor and 8.0 GB memory. For all these five algorithms, five iterations were used in the clustering stage, since only slight changes happen to the final results with more iterations, and the same post-processing procedure as declared in Section 3.3 was utilized to them.

What’s more, the distance measure for the superpixel method is different from the others; in order to fairly compare these methods with the compactness parameters in the same floating range, we have fully tested the original distance measures for all of the state-of-the-art methods and slightly tuned the distance measures of the PILS and LBSLIC to suit other methods in terms of using the same compactness parameters when conducting comparison experiments. For PILS, the dissimilarity measure was altered to *S* = *S_I_* + *C*_1_ × *m* × *S_xy_*, where *C*_1_ was set to 1/10. For LBSLIC, the dissimilarity measure was altered to *S* = *S_f_* + *C*_2_ × *m* × *S_d_*, where *C*_2_ was set to 1/3000. A 3 × 3 local patch is used in the PILS and PBSLIC methods. The 3 × 3 local target area and 9 × 9 local background regions are utilized in our GLCM method.

### 4.1. Evaluation on Simulated SAR Image

In the first stage of our experiments, the simulated SAR image is used to evaluate our proposed algorithm quantitatively. 

#### 4.1.1. Evaluation Conditions

**Figure 8:**
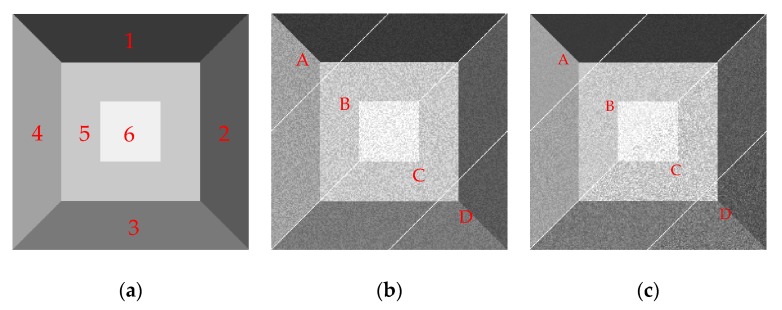
(**a**) The ground truth image; (**b**) Simulated SAR image with speckle noise variance *v* set to 0.0075, which consists of four regions i.e., A, B, C, and D; (**c**) Simulated SAR image with speckle noise variances set to 0.001, 0.004, 0.007, and 0.01 for regions A, B, C, and D, respectively.

As is shown in Figure 8, we first generate the ground truth grayscale image, which consists of 240 × 240 pixels. The image also consists of six independent regions with different gray values, and the intensities of the gray scales are 60, 90, 120, 160, 200, and 240 (based on the eight-bit single channel image) for regions one to six, respectively. Then, the MATLAB function imnoise is implemented to add multiplicative noises to the original image. As shown in Figure 8b, the image was imnoised by speckle with the noise intensity variance *v* set to 0.075. In order to show the experimental results by different parameters in a single picture, the image was divided into four regions, i.e., A, B, C, and D as shown in Figure 8b,c. Figure 8c is used to verify the robustness of the algorithms to speckles, and the variances of multiplicative noise in region A, B, C, and D are set to 0.001, 0.004, 0.007, and 0.01, respectively.

It has been widely recognized that the ability to adhere to image boundaries is the critical factor to evaluate the superpixel method [20,32]. In this article, three commonly used measures, i.e., the Boundary Recall, the Under-Segmentation Error, and the Achievable Segmentation Accuracy are used to evaluate the resulting superpixels. 

Boundary Recall (BR) [33]: BR computes the ratio of the number of pixels that the ground truth edges overlap exactly with the boundary pixels of the superpixels, that is:

(13) $$B=N_{GT\cap SP}/N_{GT},$$

where *N_GT_* is the number of boundary pixels of the ground truth, and *N_GT_*_∩*SP*_ is the number of pixels shared by the ground truth boundary and the superpixel boundary.

Under-Segmentation Error (USE) [25,34]: the ground truth segments are *R*_1_, *R*_2_, …, *R_M_*, and the superpixel results are *S*_1_, *S*_2_, …, *S_L_*; then, the USE measures how many pixels from *S_j_* “leak” across the boundary of *R_i_*. USE is defined as:

(14) $$U=\frac{1}{N}\left[\sum_{i=1}^{M}\left(\sum_{\left[S_{j}|S_{j}\cap R_{i}>B\right]}\left|S_{j}\right|\right)-N\right],$$

where |·| denotes the number of pixels, *N* denotes the total number of pixels of the image, *S_j_* ∩*R_i_* denotes the intersection of a superpixel *S_j_* with the ground truth *R_i_*, and *B* denotes the minimum number of pixels in *S_j_* overlapping *R_i_*. In this article, *B* is set to zero, which means that any pixel of a superpixel that crosses the boundary of the ground truth will be counted as an Under-Segmentation Error.

Achievable Segmentation Accuracy (ASA) [35]: ASA measures the largest overlap between superpixels and ground truth segments. ASA is defined as:

(15) $$A=\frac{\sum_{j}\max_{j}\left|S_{j}\cap R_{i}\right|}{\sum_{i}R_{i}},$$

where *S_j_* and *R_i_* are the superpixel and ground truth segment, respectively.

As is known, the quality of the resulting superpixels depends on the algorithm parameters, such as superpixel number *K*, compactness parameter *m*, and the speckle noise variance *v*. To compare our method with four existing methods on all aspects, three groups of experiments are implemented by the means of variable-controlling approach to verify the robustness of these methods to *m*, *K*, and *v*, after that, the computational efficiencies of these methods are compared.

#### 4.1.2. Robustness to Compactness Parameter *m*

For this group, we keep superpixel number *K* set to 240, and speckle noise *v* set to 0.0075. Then, superpixel methods with different compactness parameters are tested, and results with *m* set to from one to 15 are given.

**Figure 9:**
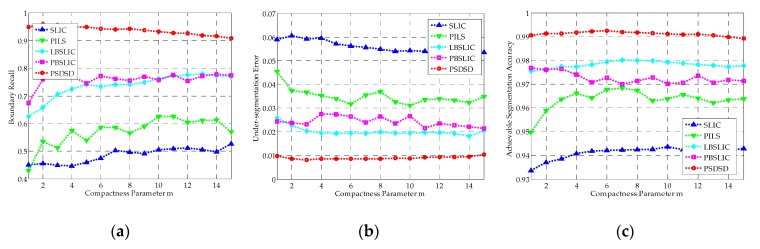
The Boundary Recall (BR), Under-Segmentation Error (USE), and Achievable Segmentation Accuracy (ASA) results for simple linear iterative clustering (SLIC), pixel intensity and location similarity (PILS), likelihood-based SLIC (LBSLIC), patch-based SLIC (PBSLIC), and pixel saliency difference and spatial distance (PSDSD) with *K* set to 240, *v* set to 0.0075, and *m* set between one and 15. (**a**) BR; (**b**) USE; (**c**) ASA.

It can be seen from Figure 9 that, in terms of BR, USE, and ASA, the SLIC method yields the worst results among these five methods; PILS performs better than SLIC, but worse than the other three methods. The BR obtained by PBSLIC is higher than LBSLIC when *m* is smaller than five, and performances of LBSLIC and PBSLIC are basically the same in terms of BR when *m* is larger than five; in terms of USE and ASA, the LBSLIC performs slightly better than PBSLIC. Overall, our proposed method is greatly superior to all the four state-of-the-art methods for any compactness parameters from one to 15 in terms of BR, USE, and ASA.

**Figure 10:**
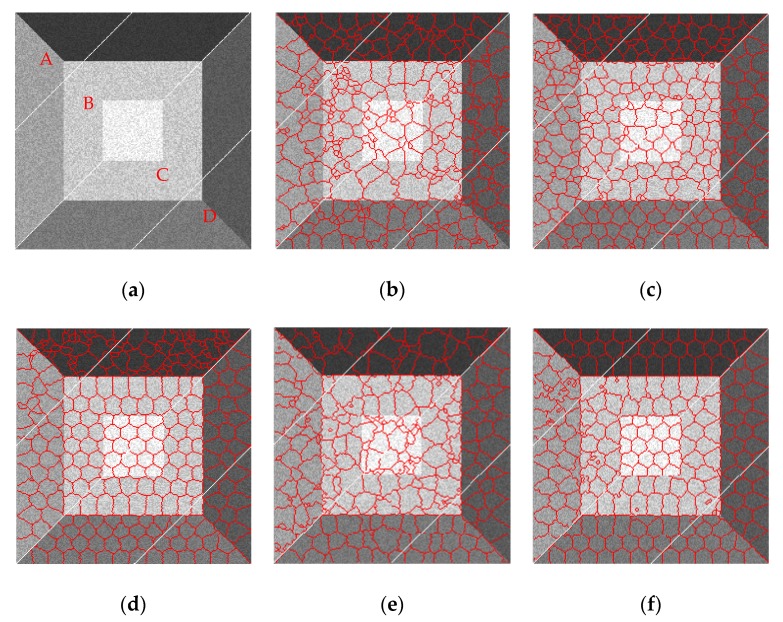
Resulting superpixels with *K* set to 240, *v* set to 0.075, and *m* set to three, six, nine, and 12 for regions A, B, C, and D, respectively. (**a**) Original; (**b**) SLIC; (**c**) PILS; (**d**) LBSLIC; (**e**) PBSLIC; (**f**) PSDSD.

As shown in Figure 10, the results obtained by SLIC can hardly adhere to the boundaries of the simulated SAR image with any compactness parameters. PILS shows a better result than SLIC, but its distance measure is not sensitive to boundaries, and a lot of wrong segments appear around the boundary regions. LBSLIC and PBSLIC show better results than PILS, but still yield bad superpixels in the boundaries of regions five and six, where pixels have lower contrasts. However, our proposed method dramatically enhances the contrasts of these boundary pixels, and obtains the best results.

#### 4.1.3. Robustness to Superpixel Number *K*

For this group, we keep the compactness parameter *m* set to eight, and speckle noise *v* set to 0.0075. Then, superpixel methods with different superpixel number *K* values are tested, and the results with *K* set to from 100 to 500 are given.

**Figure 11:**
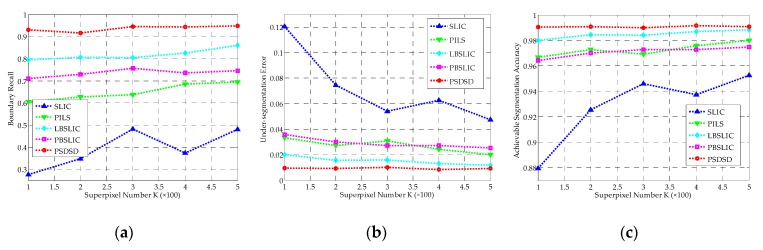
The BR, USE, and ASA results for SLIC, PILS, LBSLIC, PBSLIC, and PSDSD with *m* set to 8, *v* set to 0.0075, and *K* set to from 100 to 500. (**a**) BR; (**b**) USE; (**c**) ASA.

Under this situation, as shown in Figure 11, SLIC yields the worst results; PILS and PBSLIC perform better than SLIC, but worse than LBSLIC; our proposed method obtains the best results in terms of BR, USE, and ASA under all of the tested superpixel numbers.

**Figure 12:**
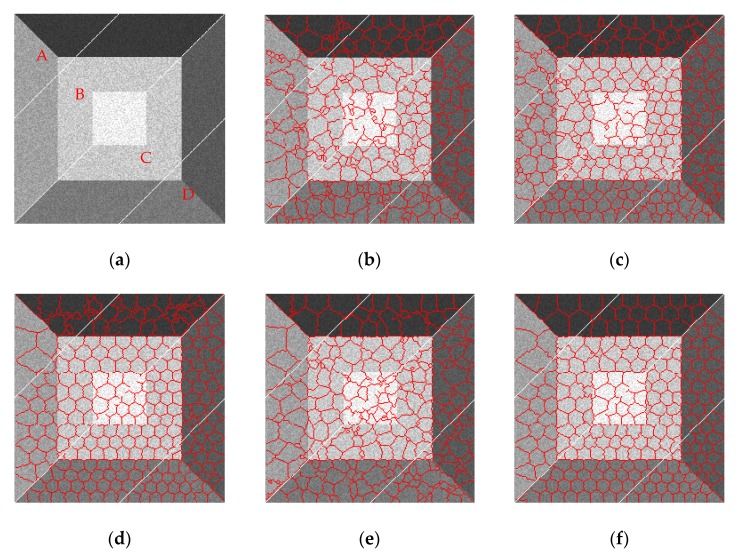
Resulting superpixels with *m* set to eight, *v* set to 0.075, and *K* set to 100, 200, 300, and 400 for regions A, B, C, and D, respectively. (**a**) Original; (**b**) SLIC; (**c**) PILS; (**d**) LBSLIC; (**e**) PBSLIC; (**f**) PSDSD.

As shown in Figure 12, similar to results in Figure 10, SLIC and PILS show poor performances in boundary adherence; LBSLIC and PBSLIC yield better results when *K* takes larger numbers such as 300 and 400, and when *K* takes small numbers, the results get worse. What’s more, for the boundaries of regions five and six, the results are still unsatisfying. Meanwhile, our method shows extraordinary results when *K* takes small numbers, and also obtains results that adhere well to the boundaries of regions five and six.

#### 4.1.4. Robustness to Speckle Variance *v*

For the third group, we keep our compactness parameter *m* set to eight, and superpixel number *K* set to 240. Then, superpixel methods with different speckle noise variance *v* are tested, and the results with speckle noise variance *v* set to from 0.001 to 0.015 are given.

**Figure 13:**
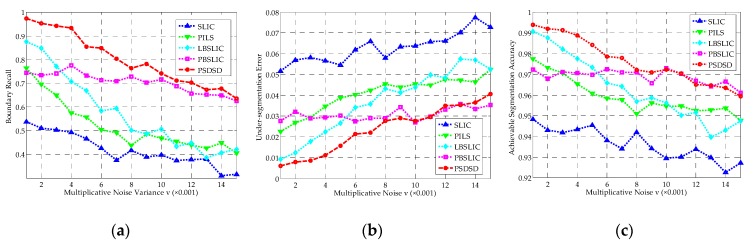
The BR, USE, and ASA results for SLIC, PILS, LBSLIC, PBSLIC, and PSDSD, with *m* set to 8, *K* set to 0.0075, and *v* set to from 0.001 to 0.015. (**a**) BR; (**b**) USE; (**c**) ASA.

The robustness of these methods to speckles are tested in this group of experiments. As shown in Figure 13, as with the increase of the noise variance, the performance of LBSLIC remarkably goes worse, and yields basically the same performance with SLIC and PILS in terms of BR. PBSLIC is inferior to LBSLIC when *v* is smaller, but its performance shows a relatively slow decline. Meanwhile, our method obtains the highest BR even under the worst condition. What’s more, in terms of USE and ASA, our method produces the same performances with PBSLIC when the speckle noise is strong.

**Figure 14:**
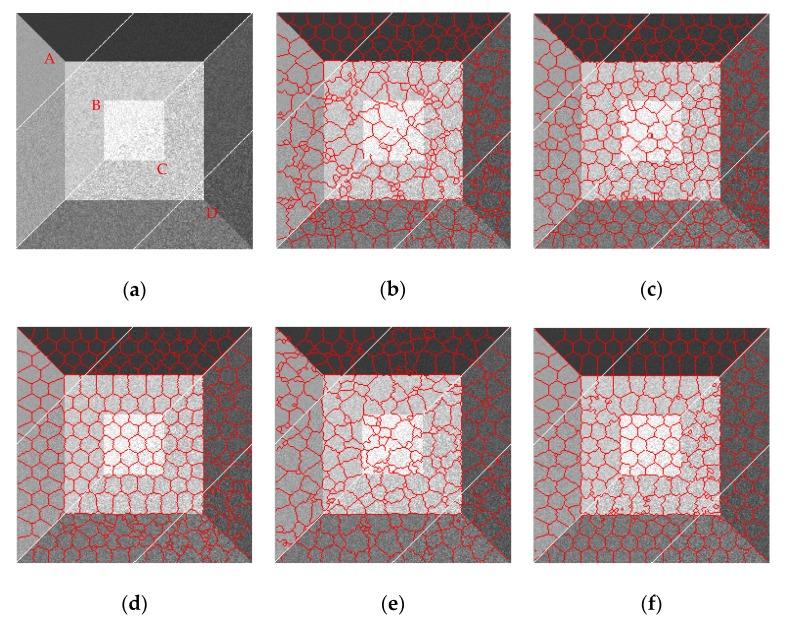
Resulting superpixels with *m* set to eight, *K* set to 240, and *v* set to 0.001, 0.004, 0.007, and 0.01 for regions A, B, C, and D, respectively. (**a**) Original; (**b**) SLIC; (**c**) PILS; (**d**) LBSLIC; (**e**) PBSLIC; (**f**) PSDSD.

From the results in Figure 14, we can see that all of these methods obtain favorable superpixel results when the noise variance is small. However, when speckles become stronger and stronger, the performances of SLIC, PILS, and LBSLIC become dramatically worse. Our method shows much better results than other methods when the speckle is relatively weaker, and in stronger noise situations, our method yields more regular results than PBSLIC.

#### 4.1.5. Computational Efficiencies

Furthermore, the segment speed of these methods is also compared. Time Cost (TC) is used to compare the segment speed of these methods. In this article, TC represents the total processing time, including the pre-processing (only our proposed method has this step, i.e., calculate the saliency map of the SAR image), the clustering and the post-processing. In our experiments, the same clustering iteration number is used, and the same post-processing procedure is adopted.

**Figure 15:**
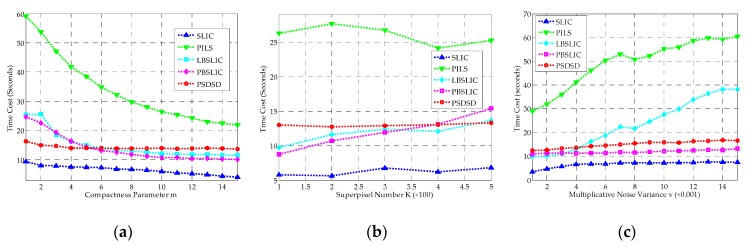
The Time Cost (TC) results for SLIC, PILS, LBSLIC, PBSLIC, and PSDSD. (**a**) Results with *K* set to 240, *v* set to 0.0075, and *m* set between one and 15; (**b**) Results with *m* set to eight, *v* set to 0.0075, and *K* set between 100 and 500; (**c**) Results with *m* set to eight, *K* set to 240, and *v* set between 0.001–0.015.

The computational efficiencies of the above experiments are shown in Figure 15. The SLIC shows fastest segment speed, while the PILS method costs at least four times that of the SLIC method for all of these experiments. Our proposed PSDSD method costs less time than LBSLIC and PBSLIC when *m* values smaller than six are used, and costs less time than LBSLIC when *v* values larger than 0.005 are used. Basically, our method only costs a little more time than LBSLIC or PBSLIC when other parameters are used. Our method costs a moderate runtime, but yields the best results.

### 4.2. Validation on Real-World SAR Images

**Figure 16:**
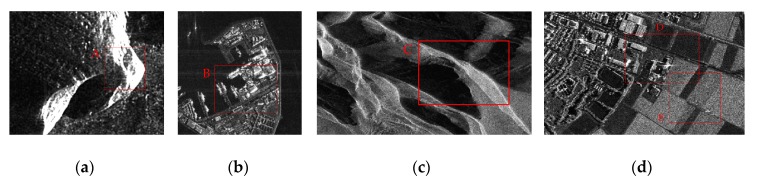
(**a**) Ayers Rock; (**b**) Ferry Port; (**c**) Antarctica; (**d**) Noerdlinger.

In this stage, the performance of our method is further evaluated by implementing experiments on four real-world SAR intensity images. As shown in Figure 16, these images were acquired by the TerraSAR-X satellite in 2007. The detailed information (including the polarization, band, size, resolution, and acquisition location) of these SAR images is listed in Table 1.

These SAR images come from different scenes. They include rocks, waters, glaciers, residential areas, and farmland. Some SAR images contain significant targets, such as rocks, ships, and buildings, while others contain textures, blocks, and other feature information. In order to better demonstrate the results of each algorithm under different parameters, we divide the image into four regions A, B, C, and D along the diagonal from top left to bottom right and implement experiments with different number of superpixels in different regions. The resulting superpixels are shown as follows.

**Figure 17:**
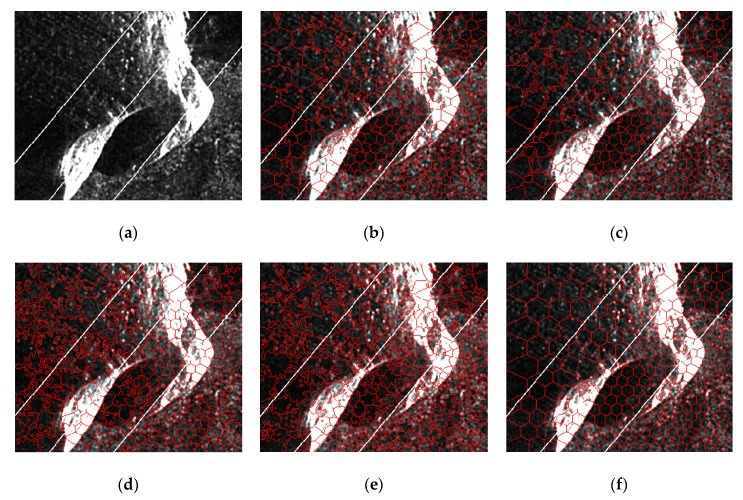
Resulting superpixels of Ayers Rock with *m* set to five and *K* set to 100, 200, 400, and 600 for regions A, B, C, and D. (**a**) Original SAR image; (**b**) SLIC; (**c**) PILS; (**d**) LBSLIC; (**e**) PBSLIC; and (**f**) PSDSD.

For the Ayers Rock, we mainly concern the boundaries of the rocks. Since there are significant rock targets in this scene, the compactness parameter is set to five, which is relatively smaller to form superpixels that adhere more to the boundaries of the targets. As we can see in Figure 17, SLIC obtains very poor results, and PILS performs better than SLIC, but still misses a lot of boundary pixels. LBSLIC and PBSLIC are sensitive to low-intensity pixels, and oversegmentations are produced in uniform regions, which are background regions. Our method yields regular superpixels in background regions, in terms of the rocks, the obtained superpixels by our method adhere well to boundaries.

**Figure 18:**
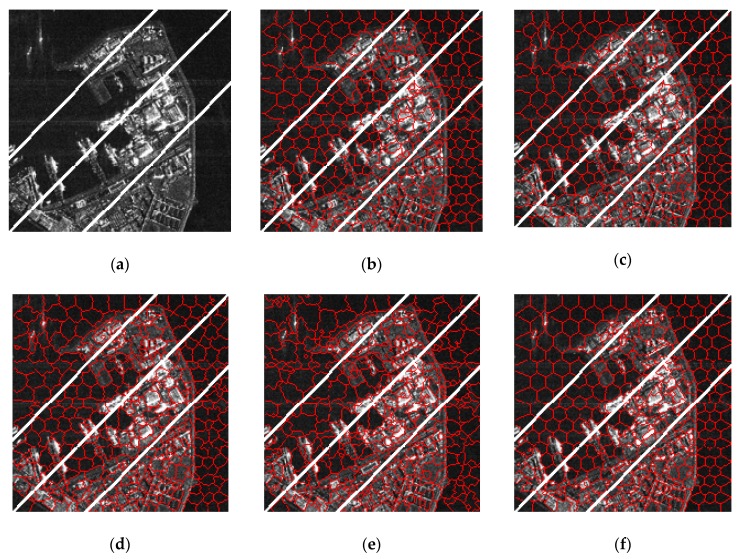
Resulting superpixels of Ferry Port with *m* set to six and *K* set to 200, 300, 350, and 500 for regions A, B, C, and D. (**a**) Original SAR image; (**b**) SLIC; (**c**) PILS; (**d**) LBSLIC; (**e**) PBSLIC; and (**f**) PSDSD.

For the Ferry Port, the results are obtained with *m* set to six, and we hope that the resulting superpixels achieve good sea land segmentation; what’s more, ship targets and buildings are well segmented. As shown in Figure 18, it is easy to see that LBSLIC, PBSLIC, and our proposed method produce superpixels that adhere to the coastline, while our PSDSD generates more regular results. Meanwhile, our method produces results when buildings have similar intensities with backgrounds and other methods don’t.

**Figure 19:**
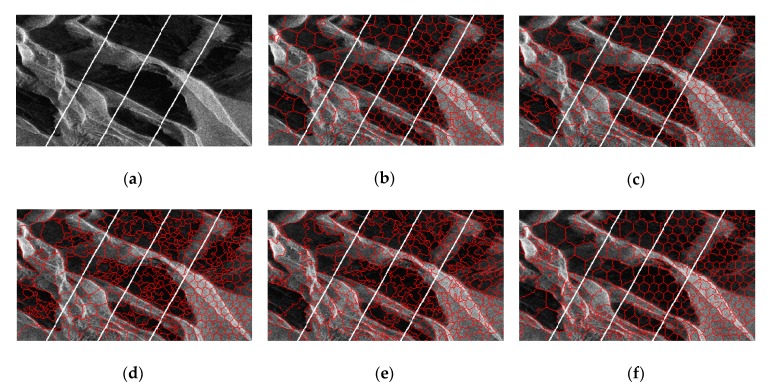
Resulting superpixels of Antarctica with *m* set to six and *K* set to 100, 200, 400, and 600 for regions A, B, C, and D. (**a**) Original SAR image; (**b**) SLIC; (**c**) PILS; (**d**) LBSLIC; (**e**) PBSLIC; and (**f**) PSDSD.

For glaciers, the results are obtained with *m* set to six, and we hope that the resulting superpixels adhere well to the texture characteristics. As shown in Figure 19, PILS, LBSLIC, and PBSLI produce many oversegmentations in shadow areas, while our method generates more regular results. In terms of the textures, our method also provides superpixels that adhere well to the glacier texture, even in textures with small changes in intensities.

**Figure 20:**
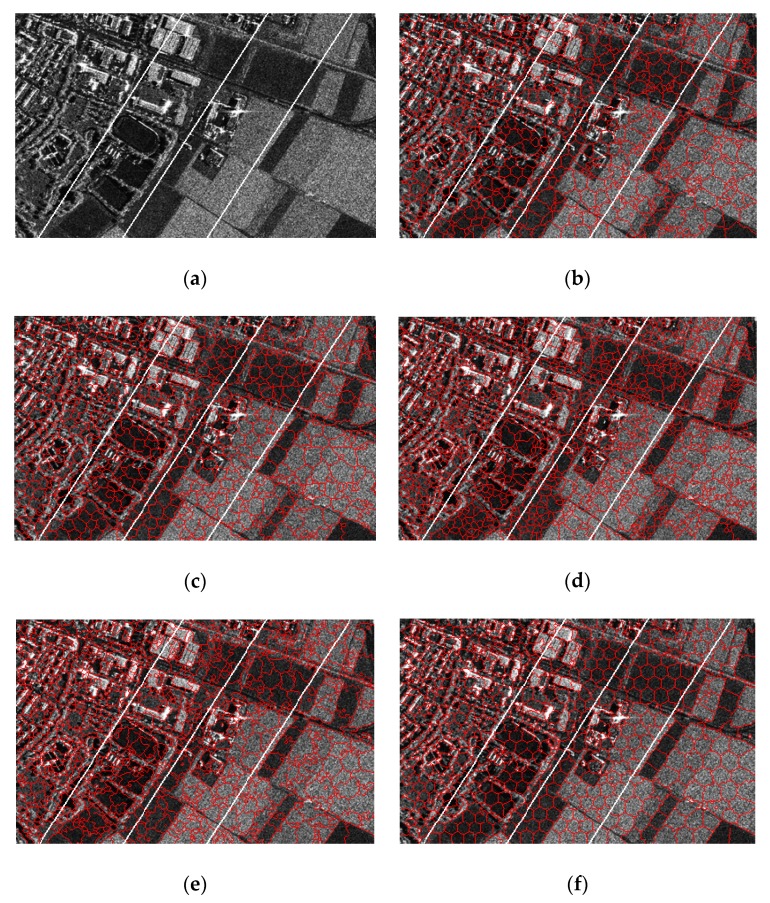
Resulting superpixels of Noerdlinger with *m* set to six and *K* set to 900, 700, 500, and 300 for regions A, B, C, and D. (**a**) Original SAR image; (**b**) SLIC; (**c**) PILS; (**d**) LBSLIC; (**e**) PBSLIC; and (**f**) PSDSD.

For hybrid scenes, traditional methods may fail to generate fine superpixels in both the target intensive areas and homogeneous regions. As shown in Figure 20, results are produced with *m* set to six. It is easy to see that, our method performs more regular superpixels in farmland, and in the residential areas, our method yields results adhere to buildings and factories. Meanwhile, results by other methods are inferior to ours.

**Figure 21:**
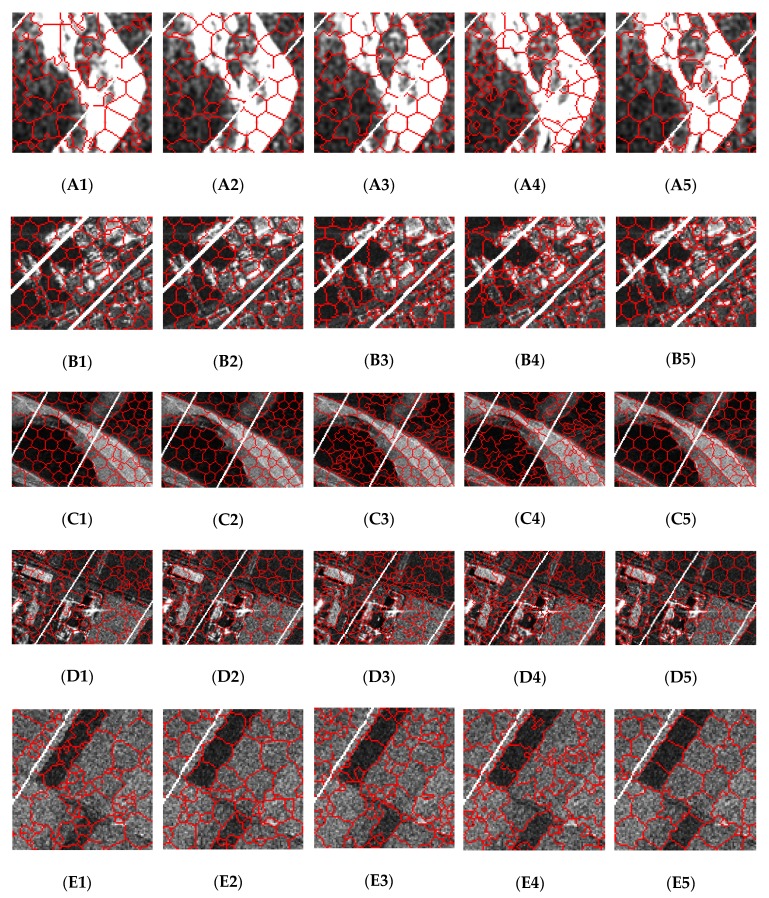
Resulting superpixels of image patches A, B, C, D, and E from the first row to the last row, respectively. The results shown from the first column to the last column are generated by SLIC, PILS, LBSLIC, PBSLIC, and PSDSD, respectively.

To further demonstrate the performance of these methods, some details in each image are shown in Figure 21. The results of image patches A, B, C, D, and E in Figure 16 are shown in the first row to the last row in Figure 21, respectively. The results shown in the first column to the last column are generated by SLIC, PILS, LBSLIC, PBSLIC, and PSDSD, respectively. It can be visually proved that our proposed method outperforms the state-of-the-art methods.

The advantages of our method are listed below by summarizing the experimental results:

First, our method has good robustness to speckles. The Gaussian kernel function is adopted to calculate the equivalent value of the pixel intensity, which can effectively suppress the speckle noise.

Second, the resulting superpixels by the proposed method show better adherence to image boundaries, especially for texture edges with intensity inhomogeneity. This is because the GLCM of the SAR image enhanced the contrast of the edge pixels, and it is easier for our method to sort these pixels to different superpixels correctly.

Third, our method has better adaptability for hybrid scenarios. Traditional methods utilize a constant compactness parameter, which turn out inappropriate results when there are regions with rich features and regions with uniform pixels in the same image. Our method utilizes an adaptive local compactness parameter; thus, regular superpixels are generated in uniform regions, and yields superpixels that adhere well to the boundaries of target structures in regions with abundant features. 

However, due to the limited space, further exploration is still needed in the future works according to the experimental results. The BR declines dramatically as with the increase of the speckle variance, although the proposed method has shown the highest BR in our evaluation experiments. Thus, the saliency map for SAR images can be further improved to produce results that are more robust to speckle noise.

## 5. Conclusions

It is difficult for the traditional superpixel methods to achieve good results for SAR images in inhomogeneous regions and hybrid regions. To solve those problems, a superpixel generating method based on pixel saliency difference and spatial distance is proposed in this article. First, a Gaussian kernel weighted LCM is introduced to calculate the saliency map of the SAR image, which can not only effectively suppress the speckle noise, but also enhance the fuzzy edges of inhomogeneous regions. Second, a computationally effective local *k*-means clustering method is used to generate superpixel results based on pixel saliency difference and spatial distance measure. In our distance measure, we utilize an adaptive local compactness parameter to adjust the weight of the saliency difference and spatial distance dynamically so as to obtain better results in hybrid senses. Third, a post-processing step is implemented to eliminate small segments. The quantitative evaluations implemented on simulated SAR images prove that our method shows a higher boundary recall and achievable segmentation accuracy and lower under-segmentation error under different parameters compared with four state-of-the-art methods. Meanwhile, the segment speed of our method is basically as fast as LBSLIC and PBSLIC, and much faster than PILS. Further, adequate experiments on real-world SAR images verified the superiority of the proposed method. Our method can effectively reduce the adverse effects of speckle noise; moreover, the results of the proposed method can adhere well to the contour of targets, and correctly reflect the boundaries of texture details, even if for the inhomogeneous regions.

## Figures and Tables

**Table 1:** Parameters for real-SAR images in Figure 16, where HH and VV denote the polarization models for horizontal transmit and horizontal receive and vertical transmit and vertical receive, respectively.

Image	Polarization	Band	Size (Pixels)	Resolution	Acquisition Location
Figure 16a	HH	X	360 × 300	1.0 m × 1.0 m	Petermann, Australia
Figure 16b	VV	X	203 × 202	7.5 m × 7.5 m	Den Helder, Nederland
Figure 16c	HH	X	355 × 198	18.5 m × 18.5 m	Ross Archipelago, Antarctica
Figure 16d	HH	X	411 × 260	3.0 m × 3.0 m	Noerdlinger, Germany

## References

[1] Li Y., Zhang Y., Yuan Z., Guo H., Pan H., Guo J. (2018). Marine Oil Spill Detection Based on the Comprehensive Use of Polarimetric SAR Data. Sustainability.

[2] Leng X., Ji K., Zhou S., Xing X., Zou H. (2017). 2D comb feature for analysis of ship classification in high-resolution SAR imagery. Electron. Lett..

[3] Dong G., Kuang G. (2015). Classification on the Monogenic Scale Space: Application to Target Recognition in SAR Image. IEEE Trans. Image Process..

[4] Wang Q., Zhu H., Wu W., Zhao H., Yuan N. (2015). Inshore ship detection using high-resolution synthetic aperture radar images based on maximally stable extremal region. J. Appl. Remote Sens..

[5] Zhao H., Wang Q., Huang J., Wu W., Yuan N. (2014). Method for inshore ship detection based on feature recognition and adaptive background window. J. Appl. Remote Sens..

[6] Xie T., Zhang W., Yang L., Wang Q., Huang J., Yuan N. (2018). Inshore Ship Detection Based on Level Set Method and Visual Saliency for SAR Images. Sensors.

[7] Leng X., Ji K., Zhou S., Xing X., Zou H. (2016). An Adaptive Ship Detection Scheme for Spaceborne SAR Imagery. Sensors.

[8] Zhai L., Li Y., Su Y. (2016). Inshore ship detection via saliency and context information in high-resolution SAR images. IEEE Geosci. Remote Sens. Lett..

[9] Yuan X., Tang T., Xiang D., Li Y., Su Y. (2014). Target recognition in SAR imagery based on local gradient ratio pattern. Int. J. Remote Sens..

[10] Xiang D., Tang T., Ni W., Zhang H., Lei W. (2017). Saliency Map Generation for SAR Images with Bayes Theory and Heterogeneous Clutter Model. Remote Sens..

[11] Benz U.C., Hofmann P., Willhauck G., Lingenfelder I., Heynen M. (2011). Multi-resolution, object-oriented fuzzy analysis of remote sensing data for GIS-ready information. Isprs J. Photogramm. Remote Sens..

[12] Moskal L.M., Styers D.M., Halabisky M. (2011). Monitoring Urban Tree Cover Using Object-Based Image Analysis and Public Domain Remotely Sensed Data. Remote Sens..

[13] Blaschke T., Hay G.J., Kelly M., Lang S., Hofmann P., Addink E. (2014). Geographic object-based image analysis—Towards a new paradigm. ISPRS J. Photogramm. Remote Sens..

[14] Heumann B.W. (2011). An Object-Based Classification of Mangroves Using a Hybrid Decision Tree—Support Vector Machine Approach. Remote Sens..

[15] Myint S., Gober P., Brazel A., Grossman-Clarke S., Weng Q. (2011). Perpixel vs. object-based classification of urban land cover extraction using high spatial resolution imagery. Remote Sens. Environ..

[16] Ren X., Malik J. Learning a classification model for segmentation. Proceedings of the IEEE International Conference on Computer Vision.

[17] Comaniciu D., Meer P. (2002). Mean shift: A robust approach toward feature space analysis. IEEE Trans. Pattern Anal. Mach. Intell..

[18] Vedaldi A., Soatto S. Quick Shift and Kernel Methods for Mode Seeking. Proceedings of the 10th European Conference on Computer Vision.

[19] Vincent L., Soille P. (1991). Watersheds in digital spaces: an efficient algorithm based on immersion simulations. IEEE Trans. Pattern Anal. Mach. Intell..

[20] Levinshtein A., Stere A., Kutulakos K.N., Fleet D.J., Dickinson S.J., Siddiqi K. (2009). TurboPixels: fast superpixels using geometric flows. IEEE Trans. Pattern Anal. Mach. Intell..

[21] Shi J., Malik J. (2000). Normalized cuts and image segmentation. IEEE Trans. Pattern Anal. Mach. Intell..

[22] Van den Bergh M., Boix X., Roig G., de Capitani B., Van Gool L. (2015). SEEDS: Superpixels Extracted Via Energy-Driven Sampling. Int. J. Comput. Vis..

[23] Xiang D., Ban Y., Wang W., Su Y. (2017). Adaptive Superpixel Generation for Polarimetric SAR Images with Local Iterative Clustering and SIRV Model. IEEE Trans. Geosci. Remote Sens..

[24] Wang W., Xiang D., Ban Y., Zhang J., Wan J. (2017). Superpixel Segmentation of Polarimetric SAR Images Based on Integrated Distance Measure and Entropy Rate Method. IEEE J. Sel. Top. Appl. Earth Obs. Remote Sens..

[25] Achanta R., Shaji A., Smith K., Lucchi A., Fua P., Süsstrunk S. (2012). SLIC Superpixels Compared to State-of-the-Art Superpixel Methods. IEEE Trans. Pattern Anal. Mach. Intell..

[26] Xiang D., Tang T., Zhao L., Su Y. (2013). Superpixel Generating Algorithm Based on Pixel Intensity and Location Similarity for SAR Image Classification. IEEE Geosci. Remote Sens. Lett..

[27] Zou H., Qin X., Zhou S., Ji K. (2016). A Likelihood-Based SLIC Superpixel Algorithm for SAR Images Using Generalized Gamma Distribution. Sensors.

[28] Yu W., Wang Y., Liu H., He J. (2016). Superpixel-Based CFAR Target Detection for High-Resolution SAR Images. IEEE Geosc. Remote Sens. Lett..

[29] Chen C.P., Li H., Wei Y., Xia T., Tang Y.Y. (2013). A Local Contrast Method for Small Infrared Target Detection. IEEE Trans. Geosci. Remote Sens..

[30] Smale S., Rosasco L., Bouvrie J., Caponnetto A., Poggio T. (2010). Mathematics of the Neural Response. Found. Comput. Math..

[31] Feng H., Hou B., Gong M. (2011). SAR Image Despeckling Based on Local Homogeneous-Region Segmentation by Using Pixel-Relativity Measurement. IEEE Trans. Geosci. Remote Sens..

[32] Veksler O., Boykov Y., Mehrani P. Superpixels and supervoxels in an energy optimization framework. Proceedings of the 11th European Conference on Computer Vision.

[33] Arbelaez P., Maire M., Fowlkes C., Malik J. (2011). Contour detection and hierarchical image segmentation. IEEE Trans. Pattern Anal. Mach. Intell..

[34] Radhakrishna A., Shaji A., Smith K., Lucchi A., Fua P., Susstrunk S. (2012). Slic Superpixels.

[35] Liu M.Y., Tuzel O., Ramalingam S., Chellappa R. Entropy rate superpixel segmentation. Proceedings of the 2011 IEEE Conference on Computer Vision and Pattern Recognition (CVPR).

